# Reduction in Soybean Flour Allergenicity Through Ball Milling Combined with γ-Aminobutyric Acid Treatment

**DOI:** 10.3390/foods14234097

**Published:** 2025-11-28

**Authors:** Lianzhou Jiang, Xiaosha Liu, Miaomiao Liu, Zhishuang Xing, Qingfeng Ban, Xiujuan Li, Zhongjiang Wang, Linyi Zhou

**Affiliations:** 1 College of Food Science, Northeast Agricultural University, Harbin 150030, China; jlzname@163.com (L.J.); lxxss0621@163.com (X.L.); liumiaomiao2023@163.com (M.L.); xingzhishuang@163.com (Z.X.); qfban@neau.edu.cn (Q.B.); 2 Heilongjiang Bingquan Duoduo Health Food Co., Ltd., Jiamusi 154000, China; bqdd8358577@126.com; 3 School of Food and Health, Beijing Technology and Business University, Beijing 100048, China

**Keywords:** soybean flour, ball milling, γ-Aminobutyric acid, antigenicity

## Abstract

Soybean flour (SF) allergy is a common food allergy reaction that significantly impacts patients’ daily diet and quality of life. This study used a combination of physical ball milling technology and γ-Aminobutyric acid (GABA) treatment to reduce the antigenicity of SF. When the material ball ratio was 1:14 (*w*/*w*), SF after ball milling treatment exhibited the smallest average particle size, and the highest solubility, bulk density, and antioxidant capacity. The functional properties of SF were further enhanced by adding GABA. Meanwhile, SF with 0.4% added GABA exhibited the smallest average particle size, the highest solubility, and the highest antioxidant capacity. The antigen content in soybean flour was determined using the soy glycinin ELISA kit and β-conglycinin ELISA kit. Compared with the original SF, the antigen contents of globulin and β-conglycinin decreased by 89.11% and 89.61%, respectively, in SF with the addition of 0.4% GABA after ball milling treatment. These results indicate that the addition of GABA not only further optimizes the solubility and antioxidant properties of SF, but also significantly reduces its antigen content. This study developed a combined treatment method to reduce allergenicity, overcoming the limitations of a single physical or biological treatment and providing a new technical approach for developing soybean flour products with low allergenicity.

## 1. Introduction

In recent years, soybean flour has been considered an ideal dairy substitute due to its absence of lactose, its cholesterol content, and its high unsaturated fatty acid content. However, compared with casein in milk, soybean flour protein also poses a risk of causing allergies. It has been reported that soybean protein allergy affects 1.9% of referred patients, with a higher incidence of allergy in pediatric patients, reaching up to 3% [1]. Some studies have also indicated that the prevalence rate may be as high as 13%. This significantly affects the application of soybean flour. Soybean flour allergies are mainly caused by soybean protein. According to the International Union of Immunological Societies (IUIS), eight allergenic proteins have been identified in soybean seeds: Gly m 1 to Gly m 8 [2]. Among them, Gly m 5 (β-conglycinin, 7S), Gly m 6 (glycinin, 11S), Gly m Bd 30K (P34), and Gly m Bd 28K (P28) are the main allergenic substances in soybean flour, particularly Gly m 5 and Gly m 6, which are highly immunogenic and represent the primary proteins responsible for eliciting IgE-mediated allergic responses in the majority of soy-bean-allergic individuals [3]. Protein allergenicity is strongly connected to structural characteristics. In recent years, with the increase in soybean flour allergies, significant progress has been made in areas such as pathogenesis, allergen identification, and treatment methods [4,5]. However, maintaining a balance between reducing the allergenicity of soybean flour and preserving its functional properties remains challenging.

Studies have reported various methods for reducing allergens in soybean flour. In the current research field, the conformational regulation of soy protein antigenic epitopes through physical methods (e.g., heating treatment, extrusion, ball milling), chemical methods (e.g., lipid–protein interaction, glycosylation), and biological methods (e.g., enzymatic catalysis, fermentation, germination) is key to reducing their antigenicity. Dong et al. [6] found that a novel thermal glycation process combining microwave treatment with sugar significantly reduced allergenicity in Atlantic cod. Cherkaoui et al. [7] reported that boiling egg whites for 45 min caused structural changes in the protein, significantly reducing its immunoreactivity and allergenicity. Most processing methods that aim to reduce soybean allergenicity often alter its physicochemical and functional properties. For example, heating treatment generally results in a reduction in nutritional value and the formation of uncontrollable harmful by-products [8]. Excessive glycosylation may lead to the formation of advanced glycation end products (AGEs), which may become new IgE binding sites, thereby increasing the allergenicity of proteins [9]. Therefore, it is of great significance to find a safe and effective method for reducing soybean allergens.

Ball milling technology, as an emerging physical modification method, has been confirmed to alter the molecular structure of proteins through mechanical force, thereby reducing their antigenicity. In recent years, ball milling treatment has shown potential in controlling the antigenicity of soybean flour. Chen et al. [10] found that ball milling treatment can alter the particle size distribution and in vitro digestion characteristics of protein, significantly reducing antigenic immunogenicity. However, improper ball milling may expose hidden linear epitopes of proteins, leading to increased allergenicity [11]. Therefore, it helps to compensate for the drawbacks of unclear modification effects through a single method using a combination of two or more modification methods to act on the protein. Ball milling combined with glycosylation modification improved the conformational rearrangement and molecular topological reconstruction of Litopenaeus vannamei protein (LVP) and its structural dynamic evolution significantly improved the colloidal stability (solubility, interfacial activity, and hydration ability) of LVP, while weakening antigen immune activity [11]. Studies reducing allergenicity by incorporating food ingredients are attracting widespread attention at present. A large number of studies have shown that the addition of bio-active small molecules can alter the structure of soy protein, thereby affecting its functional properties and allergenicity. The non-covalent complexes formed by epigallocatechin gallate with 7S and 11S globulin reduced IgE-binding ability by masking key antigenic epitopes and altering surface charge distribution and hydrophobicity [12]. γ-Aminobutyric acid (GABA) is a novel small molecule active food component that is widely present in nature. GABA plays a key role in maintaining the balance and stability of the nervous system, protecting the liver, kidneys, and intestines, and lowering blood pressure [13]. Furthermore, as an immunomodulator, GABA can suppress immune-mediated pro-inflammatory responses and has been employed in the management of various immune-related disorders in mammals [14]. Due to its immunomodulatory properties, GABA may affect the antigenicity of soybean flour. Studies have demonstrated that the addition of GABA alters the spatial conformation of soy protein isolate (SPI), leading to the disruption or masking of conformational epitopes, thereby reducing its antigenicity [15]. Therefore, the addition of GABA may mask the linear epitopes exposed by ball milling, thereby producing a synergistic effect. However, no studies have investigated the effects of ball milling combined with GABA treatment on the physicochemical properties and antigenicity of soybean flour. Therefore, investigating the physicochemical properties and allergenicity of soybean flour exposed to GABA combined with ball milling treatment is a hot research topic at present.

This study aims to investigate the effect of ball milling and ball milling combined with GABA treatment on the physicochemical properties and antigen content of soybean flour using antioxidants, dynamic light scattering, and enzyme-linked immunosorbent assays. This study may provide new ideas for the development of new, low-allergy soybean flour foods.

## 2. Materials and Methods

### 2.1. Materials

Soybean flour was procured from Heilongjiang Bingquan Duoduo Health Food Co., Ltd., Jiamusi, China. γ-Aminobutyric acid (GABA, purity ≥ 95%) was obtained from Shandong Frida Biological Co., Ltd., Jinan, China. All other chemicals used were of analytical grade.

### 2.2. Preparation of the Ball Milling Treatment

We used ball milling treatment method of Chen et al. [10] with minor modification. The soybean flour underwent ball milling treatment using a QM-3SP2 planetary ball mill (Nanjing University Instrument Factory, Nanjing, China). Two stainless steel balls (one 100 mm and one 5 mm) were put into a grinding jar. Processing was conducted under the following conditions: filling rate, 20%, rotation speed, 96 r/min, grinding time 2.5 h, and material ball ratios (*w*/*w*) of 1:8, 1:12, 1:14, 1:16 and 1:20. The treated soybean flour samples were named BSF−8, BSF−12, BSF−14, BSF−16, and BSF−20. Untreated soybean flour (SF) served as the control.

### 2.3. Preparation of the Ball Milling Combined with GABA Treatment

The GABA was added to BSF−14 to achieve final mass ratios of 1:500, 2:500, 3:500, 4:500, and 5:500 (*w*/*w*) for GABA and BSF−14. The operation parameters were chosen based on pre-experiments. Then, the complex was placed into a 16% sample solution and spray-dried by a laboratory-level spray-dryer to obtain the sample. The complex was spray-dried with the following operational settings: feed temperature of 45–55 °C, atomization rate of 300 r/s, inlet temperature of 183 °C, and outlet temperature of 80 °C [16]. The soybean flour samples were named BGSF−1, BGSF−2, BGSF−3, BGSF−4, and BGSF−5, respectively.

### 2.4. Determination of Particle Size

The particle size of soybean flour after different treatments was measured using a laser particle size analyzer (Mastersizer-3000E, Malvern Instruments Ltd., Malvern, UK). The soybean flour samples were diluted with distilled water to obtain a soybean flour concentration of 2 mg/mL. The results were expressed as the average particle size D_[4,3]_, and each sample was tested three times.

### 2.5. Determination of Bulk Density

The bulk density was determined according to the method of Jiang et al. [17] with slight modifications. A total of 1 g of soybean flour was added to a 10 mL graduated cylinder and shaken for 3 min to ensure an accurate volume measurement. The bulk density was calculated by dividing the sample’s mass by its volume. All measurements were performed in triplicate. The calculation formula is as follows:

(1) $$Bulk\;density\left(\frac{g}{mL}\right)=\frac{m_{soybean\;flour}}{V}$$

### 2.6. Determination of Wettability

A minor modification was applied to the methods described by [18] to determine the wettability. The soybean flour (0.5 g) was evenly sprinkled onto the surface of 200 mL of water maintained at 70 °C in a beaker. The time from contact with the water surface to complete wetting was recorded as the wettability time to assess the powder. Measurement was repeated three times for each sample.

### 2.7. Determination of Dispersibility

The dispersibility was measured according to Syll et al. [19] with minor modifications. Briefly, each sample (1 g) was dissolved in 10 mL of deionized water and stirred at a constant speed on a constant-temperature magnetic stirrer. The duration required to completely moisten and disperse the soybean flour clumps from the start of stirring was recorded. This assessment was repeated three times.

### 2.8. Determination of Solubility

The protein solubility was determined as described by Julakanti et al. [20] with some modifications. Soybean flour (5 g) was dissolved in 30 mL of deionized water with stirring for 30 min until complete dissolution. The solution was transferred to a 50 mL centrifuge tube, incubated in a water bath at 30 °C for 5 min, and then shaken for 3 min. The samples were centrifuged at 3000 rpm for 10 min and the supernatant was discarded. The precipitate was again centrifuged once by repeating the above operation. Finally, the precipitate was dried at 105 °C to constant weight. All measurements were performed in triplicate. The solubility of soybean flour was calculated using the following Equation:

(2) $$X\;\left(\%\right)=100-\frac{{(m}_{2}-m_{1)}\times 100}{\left(1-B\right)\times m}$$

*X* is the solubility of the sample (%); *m* is the sample mass (g); *m*_1_ is the mass of the weighing dish (g); *m*_2_ is the mass of the weighing dish and insoluble substance after drying (g); *B* is the moisture content of the sample (%).

### 2.9. Determination of Color

The color of soybean flour was measured using a color difference meter according to Drakos et al. [21], with slight modifications. White plate was taken as a standard. The color parameters were defined as follows: L* represents lightness (L* = 100 means white; L* = 0 means black), α* represents red and green (+a* means redness; −a* means greenness); and b* represents yellow and blue (+b* means yellow; −b* means blue). The measurements were taken at three randomly selected locations for each sample and averaged.

### 2.10. Determination of Macrostructure

The soybean flour obtained from different treatments was placed on black cardboard or in small bottles, and the macroscopic morphology of the samples was photographed three times using a mobile phone.

### 2.11. Determination of Microstructure

The microstructure of soybean flour was analyzed using an S-3400N SEM scanning electron microscope (SEM) (Hitachi, Ltd., Tsukuba, Japan). The sample was uniformly attached to conductive glue and had a gold layer applied to its surface. The sample was then scanned using a scanning electron microscope at an acceleration voltage of 5.0 kV and observed at magnifications of 5 k. Determination of particle size distribution in microscopic images using image measurement software (Nano Measurer V1.2, Fudan University, Shanghai, China).

### 2.12. Determination of Viscosity

The soybean flour’s viscosity was measured using a plate measurement system (model P35, diameter 35 mm) with a plate spacing of 1 mm at 25 °C. The shear rate increased from 0.1 to 500 s^−1^. Each soybean flour sample was measured three times.

### 2.13. Determination of Dynamic Rheology

The dynamic rheology of the soybean flour was measured using a plate measurement system (model P35, diameter 35 mm) with a plate spacing of 1 mm at 25 °C. The changes in storage modulus (G′) and loss modulus (G″) with frequency were measured under the setting conditions of strain force of 1% and oscillation frequency of 0.1–10 Hz. Each soybean flour sample was measured three times.

### 2.14. Determination of DPPH Radical Scavenging Activity

The DPPH radical scavenging activity was based on the method proposed by Liu et al. [22] and was modified accordingly. The sample solution was mixed with twice the volume of DPPH solution and incubated in the dark at room temperature for 30 min. Then, the absorbance of the mixture at 517 nm was measured using an ultraviolet spectrophotometer. All measurements were performed in triplicate. The DPPH radical scavenging activity was calculated as follows:

(3) $$DPPH\left(\%\right)=\left(1-\frac{A_{i}-A_{j}}{A_{c}}\right)\times 100\%$$

where *A_i_* is the absorbance value after mixing the sample solution with DPPH solution; *A_j_* is the absorbance value of the sample solution mixed with ethanol; and *A_c_* is the absorbance value of ethanol mixed with DPPH solution.

### 2.15. Determination of ABTS Radical Scavenging Activity

The ABTS radical scavenging activity of cottonseed peptides was determined according to the method of Wang et al. [23] with minor modifications. The ABTS working solution was prepared by mixing 7 mmol/L ABTS with an equal volume of 2.45 mmol/L potassium persulfate, followed by incubation in the dark at room temperature for 12–16 h. This working solution was diluted with anhydrous ethanol to an initial absorbance (*A*_0_) of 0.70 ± 0.03 at 734 nm. Subsequently, 50 μL of the sample solution was mixed with 3 mL of the working solution and incubated in darkness at 25 °C for 6 min to measure the absorbance (*A*). All measurements were performed in triplicate. The ABTS scavenging activity was calculated as follows:

(4) $$ABTS\left(\%\right)=\left(1-\frac{A}{A_{0}}\right)\times 100\%$$

### 2.16. Determination of Antigen Content

The antigen content of the sample was determined using soy glycinin ELISA kit and β-conglycinin ELISA kit (Shanghai Jianglai Biotechnology Co., Shanghai, China). The method was adapted from the approach outlined by Xing et al. [15] with simple modifications. A 0.3 g sample was mixed with 30 mL of 1× sample extraction working solution. After shaking in a 37 °C water bath, the mixture was centrifuged (4000× *g*, 25 °C, 5 min), and the supernatant was diluted. The test strip was sequentially filled with 50 μL per well of the calibration/test sample and antibody working solution, then incubated for 10 min. After drying the washed plate, the enzyme-labeled reagent, the color developing solution, and the termination solution were sequentially added. The absorbance was read at 450 nm and 630 nm using the Microplate reader (SpectraMax reg iD3, Molecular Devices, South San Francisco, CA, USA). All measurements were performed in triplicate.

### 2.17. Statistical Analysis

Each experiment was performed three times, and all results were presented as mean ± standard deviation. Means were compared using Duncan’s test (*p* < 0.05) after a one-way analysis of variance (ANOVA), utilizing SPSS Statistics 26.0 software.

## 3. Results

### 3.1. The Particle Size of BSF Under Different Material Ball Ratios

**Figure 1:**
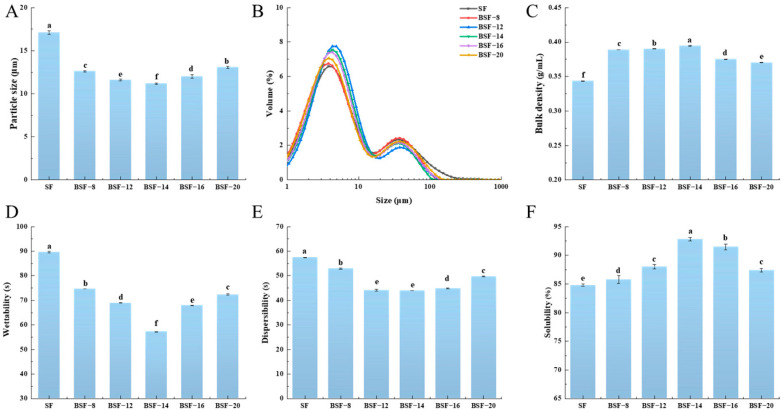
Particle size (**A**), particle size distribution (**B**), bulk density (**C**), wettability (**D**), dispersibility (**E**) and solubility (**F**) of soybean flour (SF) and soybean flour treated by ball milling at different material ball ratios (BSF). All results are presented as mean ± standard deviation (*n* = 3). Different letters in each column represent significant differences (*p* < 0.05).

The particle size of soybean flour powders has a significant impact on its sensory texture. Large particle sizes often lead to a rough taste, which may reduce consumer acceptance [21]. As shown in Figure 1A, the particle sizes of soybean flour after ball milling treatment at different material ball ratios were investigated. SF had the largest particle size, which was 17.10 μm. The particle size of the soybean flour powders significantly decreased after ball milling treatment (*p* < 0.05). The particle size of BSF showed a trend of initially decreasing and then increasing with increases in the material ball ratio (*w*/*w*). This study indicated that the material ball ratio has a significant effect on the particle size of BSF. When the material ball ratio was 1:14 (*w*/*w*), BSF had the smallest particle size, which was 11.17 μm. This may be due to the fact that, at this material ball ratio, the ball milling treatment provided high grinding efficiency and increased the frequency of collisions among particles, thereby promoting further refinement of the particle size. This was consistent with the work of Ahmed et al. [24], who found that ball milling could significantly reduce the particle size of foxtail millet flour. Reducing the particle size of soybean flour can improve its functional properties, such as wettability, dispersibility, and solubility. Furthermore, the reduction in particle size and increase in the specific surface area of soybean flour induced by ball milling promotes the unfolding of the protein structure. This structural alteration disrupts conformational epitopes and may modify the accessibility of linear epitopes, thereby leading to a reduction in allergenicity [25].

Particle size distribution (PSD) is a critical parameter affecting powder properties such as bulk density and flowability, playing a vital role in industrial applications [26]. Figure 1B shows the particle size distribution of BSF at different material ball ratios. The particle size of BSF has a bimodal distribution. The particle sizes of BSF were mainly concentrated in the range of 1–10 μm, with a small number of particles distributed between 10 and 100 μm. Compared with SF, BSF exhibited a narrower particle size distribution peak (1–10 μm) and a higher volume percentage. Additionally, the particle size distribution shifted to the left in the range of 10–100 μm. When the material ball ratio was 1:14, this phenomenon was most significant. This indicates that the particle size distribution of the sample was relatively uniform and mainly concentrated in a narrow range. After sufficient grinding treatment, soybean flour can be effectively broken down into finer particles. This reduction in particle size not only improves key properties of the soybean flour solution, such as wettability, dispersibility, and solubility, but also further enhances the stability of soybean flour products.

### 3.2. The Bulk Density of BSF Under Different Material Ball Ratios

Bulk density means the mass of flour per unit volume, which is usually influenced by factors such as particle morphology, particle size, particle size distribution, and interactions between particles [27]. As shown in Figure 1C, soybean flour without ball milling treatment had the lowest bulk density, which was 0.34 g/mL. After ball milling treatment, the bulk density of BSF significantly increased (*p* < 0.05), and the bulk density of BSF rose at first and then declined with the increase in material ball ratio. This may be due to the reduction in the particle size of soybean flour caused by ball milling treatment. This results in the soybean flour becoming tightly packed, leading to an increase in bulk density [28]. When the material ball ratio was 1:14, the bulk density of BSF reached its maximum value, which was 0.39 g/mL. This indicated that ball milling treatment is involved in the grinding process, enhancing the impact and friction of material particles and thereby increasing the bulk density. Appropriately increasing the bulk density of soybean flour can prevent agglomeration and oxidation among soybean flour particles, which improves the solubility and flowability of soybean flour. However, when the material ball ratio is too high, this may lead to the excessive refinement and fragmentation of particles, causing particle agglomeration. This can affect the flowability and bulk density of the soybean flour [29]. This finding was consistent with that of Gong et al. [27], who found that long-term cavitation jet treatment caused a reduction in the particle size of soymilk flour. Fine particles tend to adhere to each other, hindering efficient filling.

### 3.3. The Wettability of BSF Under Different Material Ball Ratios

Wettability is usually determined by the surface energy, particle morphology, and intermolecular forces of powder particles [30]. Wettability directly affects the dissolution rate, dispersion uniformity, and stability of soybean flour in liquids, which are crucial for the quality of food processing. As shown in Figure 1D, the wettability of BSF was investigated at different material ball ratios. SF had the longest wetting time, which was 89.61 s. After ball milling treatment, the wetting time of soybean flour was significantly reduced (*p* < 0.05). As the material ball ratio increased, the wetting time showed a trend of an initial increase followed by a decrease. When the material ball ratio was 1:14, the wettability of soybean flour was the highest. This may be due to ball milling causing the surface of soybean flour particles to become rough and the specific surface area to increase, thereby increasing the surface energy. This increase in surface energy can promote rapid adsorption and increased contact with water molecules, thus leading to relatively strong wettability in the initial wetting stage. However, an excessively high material–ball ratio can lead to excessive grinding, causing particle aggregation and agglomeration, and thereby reducing surface energy and affinity with water molecules. This usually manifests through a prolonged wetting time and decreased wettability. This finding is consistent with that of Ren et al. [18], who found that extended enzymatic hydrolysis may lead to aggregation among soy proteins. This aggregation masked the soy lecithin’s binding sites, reduced structural flexibility, and hindered interactions with water. This led to an increase in wetting time.

### 3.4. The Dispersibility of BSF Under Different Material Ball Ratios

Dispersibility refers to the dispersion rate and stability of powder particles in a liquid. In the food industry, excellent dispersibility ensures that soybean flour products form a homogeneous suspension without sedimentation, which is critical for product stability, shelf life, and consumer acceptance [31]. As shown in Figure 1E, SF had the longest dispersion time in liquid, which was 57.40 s. This indicates that soybean flour had the worst dispersibility at this time. After ball milling treatment, the dispersibility of soybean flour was significantly improved (*p* < 0.05). As the material ball ratio increased, the dispersibility decreased at first and then increased. When the material ball ratio was 1:14, BSF−14 had the shortest dispersion time of 43.95 s, indicating that BSF−14 possessed the highest dispersibility. At this time, BSF−14 had the smallest particle size. This increased the contact area and contact points between the particles and water, thereby enhancing dispersibility. However, excessive ball milling not only reduces particle size, but may also cause surface cracking and the adhesive aggregation of particles. This agglomeration reduces the flowability and dispersibility of the powder in liquids and increases the viscosity of the dispersion, thereby prolonging the dispersion time of soybean flour in liquids [32].

### 3.5. The Solubility of BSF Under Different Material Ball Ratios

The solubility of soybean flour is one of the key factors affecting its dispersibility and application in liquids, and is of great significance in food processing and nutrient absorption [27]. As shown in Figure 1F, SF had the lowest solubility, which was 84.79%. After ball milling treatment, the solubility of soybean flour significantly increased (*p* < 0.05). This may be because ball milling destroys the surface structure and crystalline zone of soybean flour particles through mechanical force, resulting in surface roughness and a reduction in particle size. Such changes in structure and size increase the particles’ specific surface area and degree of disorder, thereby significantly enhancing the solubility of soybean flour. In addition, after ball milling, the polar groups inside the protein are exposed, the surface charge of the protein molecules changes, and the hydration effect is enhanced. The solubility of soybean flour exhibited a trend of an initial increase followed by a decrease as the material ball ratio rose. When the material ball ratio was 1:14, BSF−14 had the highest solubility, which was 92.78%. This may be due to the impact of grinding media (such as steel balls) on the sample, causing the soybean flour particles to be broken into fine particles. These fine particles tend to come into contact with the solvent and dissolve quickly [33]. At the same time, the structure and connections between particles will also change, ultimately leading to an increase in solubility.

### 3.6. The Rheological Characterization of BSF Under Different Material Ball Ratios

**Figure 2:**
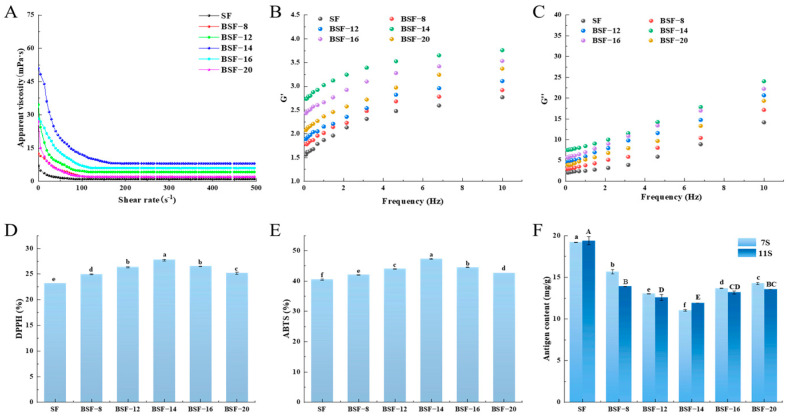
Apparent viscosity (**A**), storage modulus (G′) (**B**), loss modulus (G″) (**C**), DPPH (**D**), ABTS (**E**), and antigen content (**F**) of soybean flour (SF) and soybean flour treated by ball milling at different material ball ratios (BSF). All results are presented as mean ± standard deviation (*n* = 3). Different letters in each column represent significant differences (*p* < 0.05). Capital letters indicate significant differences in 11S content, while lowercase letters indicate significant differences in 7S content (*p* < 0.05).

Apparent viscosity is an important indicator for measuring the flow resistance of liquids and is closely related to the mouthfeel of soybean flour. An increase in the viscosity of soybean flour usually results in a thick and smooth sensation in the mouth [34]. The viscosity of BSF at different material ball ratios is shown in Figure 2A. All samples showed non-Newtonian fluid behavior, with shear viscosity decreasing as shear rate increased and exhibiting shear thinning pseudoplastic fluid phenomenon. The viscosity of SF was the lowest. As the material ball ratio increased, the viscosity first increased and then decreased and reached its maximum value at a ratio of 1:14. This may be because, as ball milling time and intensity increased, soy protein and fat particles in the soybean flour solution were broken down into smaller particles, leading to changes in their surface properties. These fine particles enhance the dispersion of the liquid and may form a compact fluid structure, thereby increasing the viscosity of the soybean flour solution. Jiang et al. [35] also confirmed that the ball milling treatment significantly reduced particle size and strengthened the intermolecular force, thereby leading to an increase in apparent viscosity. However, when the material ball ratio was excessively high, the apparent viscosity of BSF decreased. Excessive ball milling treatment may lead to the degradation of proteins and fats in soybean flour, resulting in a loss of partial structures and a consequent decrease in viscosity.

The relationships between the storage modulus (G′) and loss modulus (G″), as a function of frequency for soybean flour solutions treated with different material ball ratios, are shown in Figure 2B,C. It can be seen that both G′ and G″ of SF were the lowest. After ball milling, both G′ and G″ of BSF increased significantly, and throughout the entire frequency range, G′ was less than G″. This suggests a liquid-like flow behavior in the soybean flour solution. This may be due to the ball milling treatment altering the viscoelastic properties of the soybean flour; therefore, many of the nutritional components in soybean flour (such as protein, fat, and dietary fiber) are released [36]. With the increase in material ball ratio, both G′ and G″ of BSF showed a trend of first increasing and then decreasing. When the material ball ratio was 1:14, the G′ and G″ values of BSF−14 reached their maximum. This phenomenon further confirms that soybean flour solution prepared at a material ball ratio of 1:14 exhibits superior flowability compared to other processing conditions. Additionally, the possibility of soybean flour agglomeration was significantly reduced, and the highest dispersibility and solubility were obtained.

### 3.7. The Antioxidant Capacity of BSF Under Different Material Ball Ratios

The antioxidant capacity is one of the main functional characteristics exhibited by soybean flour. The DPPH and ABTS free radical scavenging rates were used to evaluate the effect of ball milling on the antioxidant capacity of soybean flour. As shown in Figure 2D,E, the antioxidant capacity of SF was the lowest, and its DPPH and ABTS free radical scavenging rates were 23.23% and 40.49%, respectively. After ball milling treatment, the free radical scavenging ability of soybean flour was significantly improved (*p* < 0.05). This may be to the mechanical force generated by ball milling breaking down the cell walls of soybean flour and releasing antioxidant active substances such as soy isoflavones and polyphenols, which in turn inhibit and scavenge free radicals [37,38]. This structural breakdown also reduces particle size and increases surface area, which further enhances the release of these bioactive compounds [39]. The antioxidant capacity of BSF first increased then decreased with increasing material ball ratio, reaching its maximum at a material ball ratio of 1:14. This may be because soybean flour had the smallest particle size at this material ball ratio. This reduced size facilitated the most effective release of bioactive compounds, consequently leading to the highest antioxidant capacity. Hu et al. [40] also confirmed that superfine grinding treatment can reduce the particle size of green tea flour, thus enhancing its antioxidant activity.

### 3.8. The Antigen Content of BSF Under Different Material Ball Ratios

Soybean flour allergies are primarily caused by soy protein, with 7S and 11S being the main allergenic substances found in soybean flour. The antigen content of soybean flour after ball milling is shown in Figure 2F. The antigen contents of 7S and 11S of SF were the highest, at 19.20 mg/g and 19.39 mg/g, respectively. After ball milling treatment, the antigen contents of 7S and 11S of BSF significantly decreased. When the material ball ratio was 1:14, the antigen contents of 7S and 11S in BSF were the lowest, at 11.02 mg/g and 11.91 mg/g, respectively. On the one hand, the decrease in the antigen content might be associated with the conformational changes in or destruction of the linear epitopes of globulin molecules caused by ball milling treatment [41]. On the other hand, the intense mechanical forces, such as shear, vibration, and friction, during ball milling may have disrupted some of the globulin’s IgE binding epitopes, thereby reducing their capacity to bind to the ELISA detection antibodies. This finding was consistent with the work of Chen et al. [10], who found that ball milling treatment significantly reduced the content of tropomyosin in natural oyster protein (OP) from 19.56 ng/g to 17.80 ng/g, as well as decreasing the allergenicity of OP by 9.00%. Therefore, ball milling treatment can reduce the antigenicity of soybean flour; BSF−14 exhibited the lowest antigen content.

However, although the antigen content in soybean flour decreased, it remained at a certain level. This did not achieve the expected effect of reducing allergenicity. Therefore, we adopted a treatment combining ball milling with GABA to achieve the goal of producing hypoallergenic products.

### 3.9. The Particle Size of BGSF Under Different Concentrations of GABA

**Figure 3:**
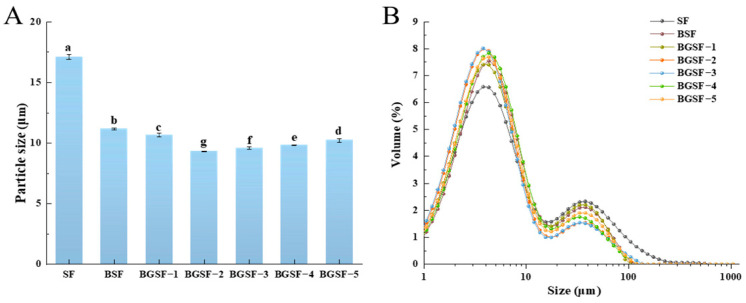
Particle size (**A**) and particle size distribution (**B**) of soybean flour (SF), soybean flour treated by ball milling (BSF), and soybean flour that underwent ball milling combined with GABA treatment (BGSF). All results were presented as mean ± standard deviation (*n* = 3). Different letters in each column represent significant differences (*p* < 0.05).

The particle size of soybean flour powders after the ball milling combined with GABA treatment is shown in Figure 3A. After the ball milling combined with GABA treatment, the particle size of BGSF was significantly further reduced (*p* < 0.05), showing an initial decrease followed by an increase. The BGSF−2 had the smallest particle size, at 9.33 μm. This may be because proper spray-drying parameters can effectively reduce the particle size of soybean flour. Additionally, GABA may interact with the protein to inhibit its coagulation and gelation, thus affecting the particle size of soybean flour after spray-drying [15]. Cui et al. [42] also confirmed that the incorporation of GABA reduced the mean particle size of SPI compared to SPI without GABA. This confirmed that the ball milling combined with GABA treatment significantly reduces the particle size of BGSF, and this combination treatment method is more effective than a single treatment method.

From Figure 3B, it can be clearly observed that all samples exhibit a bimodal distribution and the particles of the soybean flour solution were mainly concentrated in the range of 1–10 μm. The particle size distribution of the BGSF−2 was the most concentrated, presenting a distinct “thin and tall” shape. This means that most of the soybean flour particle sizes were mainly concentrated in a smaller size range. This result further confirmed that the ball milling combined with GABA treatment can improve the particle distribution of soybean flour powders.

### 3.10. The Bulk Density of BGSF Under Different Concentrations of GABA

**Figure 4:**
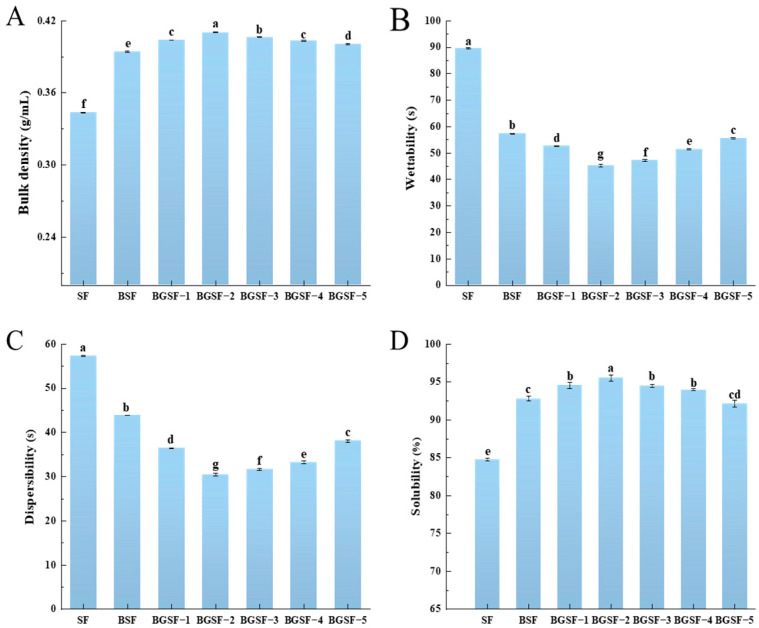
Bulk density (**A**), wettability (**B**), dispersibility (**C**) and solubility (**D**) of soybean flour (SF), soybean flour treated by ball milling (BSF) and soybean flour that underwent the ball milling combined with GABA treatment (BGSF). All results are presented as mean ± standard deviation (*n* = 3). Different letters in each column represent significant differences (*p* < 0.05).

As shown in Figure 4A, the ball milling combined with GABA treatment further improved the bulk density of BGSF, showing an initial increase and a subsequent decrease. The BGSF−2 had the highest bulk density, at 0.41 g/mL. On the one hand, the water evaporation during spray-drying reduces the space between soybean flour particles [43]. On the other hand, the addition of GABA may change the surface properties of soybean flour particles, enhancing the interactions between particles and thereby increasing the bulk density. This finding is consistent with the work of Fernandes et al. [44], who found that rosemary essential oil applied using the spray-drying method increased the encapsulation concentration, leading to an increase in the bulk density of the samples. The above results indicate that the synergistic effect of the two treatments significantly increases the bulk density of soybean flour compared with single ball milling treatment. For soybean flour products, increasing the bulk density can improve the flowability of soybean flour particles and, to a certain extent, enhance their solubility and dispersibility. Therefore, the ball milling combined with GABA treatment can effectively increase the bulk density of soybean flour to improve its processing performance.

### 3.11. The Wettability of BGSF Under Different Concentrations of GABA

As shown in Figure 4B, SF had the longest wetting time. This may be attributed to its high particle density, low specific surface area, and strong surface hydrophobicity. After the ball milling combined with GABA treatment, the wetting time of BGSF showed a trend of first decreasing and then increasing. This may be due to the low concentration of GABA molecules that are able to form a hydrophilic layer on the surface of soybean flour, enhancing the interaction between soybean flour and water [14]. BGSF−2 had the shortest wetting time and the highest wettability. This indicates that a synergistic effect is generated between ball milling treatment and the addition of GABA, further enhancing the hydrophilicity of soybean flour. Overall, ball milling treatment disrupts the particle structure of soybean flour. On the one hand, this increases the surface roughness of the particles; on the other hand, it promotes the dispersion and adsorption of GABA. These changes have a synergistic effect, ultimately improving the wettability of soybean flour. This improvement enhances the bioavailability of nutrients (e.g., proteins, fatty acids, minerals) in soybean flour, thereby facilitating their absorption by the human body.

### 3.12. The Dispersibility of BGSF Under Different Concentrations of GABA

The dispersibility of powder products is one of their most critical characteristics. High-quality soybean flour particles can dissolve independently in water, without aggregation. Thus, improving dispersibility is crucial for the industrial application and consumer acceptance of soybean flour products [45]. As shown in Figure 4C, SF had the longest dispersion time in liquid. This indicates that soybean flour had the worst dispersibility at this time. After ball milling treatment, the dispersibility of BSF was improved. The addition of GABA further improved the dispersibility of soybean flour, and showed a trend of first decreasing and then increasing with the addition of GABA. This may be because the addition of an appropriate amount of GABA changes the surface properties of soybean flour particles, which enhances the repulsive forces between the particles and thereby reduces the agglomeration phenomenon. Among all samples, BGSF−2 exhibited the highest dispersibility. This may be attributed to the adsorption and distribution of GABA on soybean flour particles; an appropriate amount of GABA can optimize the interactions between particles, thereby achieving the effect of improving dispersibility [15]. Excessive or insufficient GABA can lead to a decrease in dispersibility. The above results indicate that the ball milling combined with GABA treatment can further reduce the dispersion time of soybean flour in liquid and improve its dispersibility on the basis of a single treatment.

### 3.13. The Solubility of BGSF Under Different Concentrations of GABA

As shown in Figure 4D, the solubility of BGSF at different concentrations of GABA was investigated. Compared with a single ball milling treatment, the ball milling combined with GABA treatment produced a synergistic effect, further improving the solubility of soybean flour. With an increase in the concentration of GABA, the solubility of soybean flour showed an initial rise followed by a decline. The solubility reached its maximum in BGSF−2, at 95.56%. On the one hand, the hydrophilicity of GABA effectively improves the hydration of soybean flour, resulting in the enhancement of solubility [46,47]. On the other hand, the interactions between GABA and the surface of soybean flour increase the hydrophilicity of soybean flour particles, thereby facilitating the penetration of water into the powder. Furthermore, ball milling enhanced soybean flour solubility by refining particles, increasing surface area, and improving surface structure [32]. The synergistic effect of ball milling and GABA significantly improved the solubility of soybean flour particles by optimizing their physical properties and surface characteristics. Higher protein solubility facilitates greater accessibility to digestive enzymes, thereby promoting enzymatic hydrolysis. This may reduce the allergenic risk, as short peptide fragments and amino acids are generally non-allergenic [15].

### 3.14. The Color of BGSF Under Different Concentrations of GABA

As shown in Table 1, the chromaticity values (L*, a* and b* value) of BGSF at different concentrations of GABA were investigated. After ball milling treatment, the L* value of BSF significantly increased, to 84.72. This may be due to a change in moisture during the ball milling process, which leads to a bright color of the sample. This result is consistent with that of Takahashi et al. [48], who found that ultracentrifuge cryomilling technology can grind rice flour to the micron level. This process not only significantly reduces the particle size of rice flour, but also unexpectedly increases its brightness value. The a* and b* values of BSF also significantly increased, to 2.56 and 28.41, respectively. This color change is closely related to the pigment content in soybean flour. The natural pigments in soybean flour, such as anthocyanins and carotenoids, may be released or activated through mechanical force during ball milling, leading to an increase in a* value [21]. The ball milling combined with GABA treatment had a significant effect on the L*, a*, and b* values of BGSF. The L* value displayed an initial increase followed by a subsequent decrease, and the BGSF−2 had the highest L* value, which was 86.56. This indicated that after adding GABA, the color of soybean flour may lean towards light yellow. The a* and b* values of BGSF showed a gradually decreasing trend. On the one hand, the mechanical force generated during ball milling may damage the structure and crystalline structure of starch particles, resulting in smaller starch particles and rougher surfaces in soybean flour. Meanwhile, the polarization cross phenomenon gradually disappears, which in turn affects the color [48]. On the other hand, during the process of spray-drying, nozzle atomization reduces particle size and alters aggregation, thereby influencing soybean flour’s color.

### 3.15. The Morphological Characteristics of BGSF Under Different Concentrations of GABA

**Figure 5:**
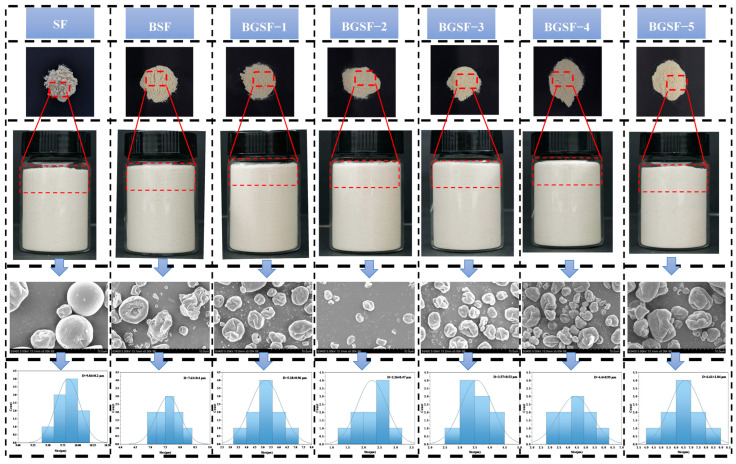
Morphological characteristics of soybean flour (SF), soybean flour treated by ball milling (BSF), and soybean flour under the ball milling combined with GABA treatment (BGSF).

The morphological characteristics of soybean flour treated with the ball milling combined with GABA treatment are shown in Figure 5. From the macroscopic image of soybean flour, it can be seen that SF is relatively loose, with an uneven particle size and local aggregation phenomenon. Compared with SF, the texture of BSF became finer and the aggregates decreased after ball milling treatment. This may be due to the frictional force, impact force, and shear force generated by ball milling acting together on the surface of soybean flour particles, resulting in particle breakage and refinement [49]. After the addition of GABA, the particle size of BGSF was further reduced. Among all samples, BGSF−2 had the smallest particle size, the most compact particle state, and the least aggregation.

Scanning electron microscopy (SEM) was used to observe the microstructure of soybean flour after different treatments. SF had a smooth surface [25]. After ball milling treatment, the particle size of BSF decreased, and surface cracks and damage appeared. This may be due to the continuous action of collision, friction, and shear force between the balls and soybean flour causing the soybean flour particles to reduce in size. Additionally, the mechanical forces cause partial disruption and denaturation of the protein surfaces, resulting in depressions and cracks. These morphological changes increase the exposed surface area of proteins, enhance their interactions with water or other components, and thereby improve solubility [50]. After adding GABA, the aggregation phenomenon of BGSF particles was significantly reduced. The surface roughness of the BGSF particles decreased, and the depression phenomenon was reduced. Through a quantitative analysis of particle size distribution from micrographs, it can be observed that with the increase in GABA content, the size of soybean flour particles shows a trend of first decreasing and then increasing. In BGSF−2, soybean flour particles significantly decreased in size. This phenomenon is consistent with the results for particle size. The results further confirmed that the ball milling combined with GABA treatment can effectively reduce the particle size of soybean flour, which helps to improve the solubility and dispersibility of soybean flour, thereby enhancing its commercial application value.

### 3.16. The Rheological Characterization of BGSF Under Different Concentrations of GABA

**Figure 6:**
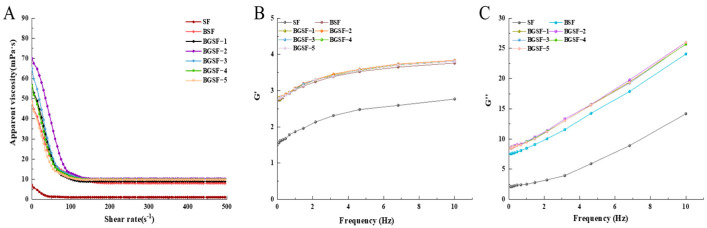
Apparent viscosity (**A**), storage modulus (G′) (**B**), and loss modulus (G″) (**C**) of soybean flour (SF), soybean flour treated by ball milling (BSF), and soybean flour under the ball milling combined with GABA treatment (BGSF).

As shown in Figure 6A, all samples showed a trend of decreasing apparent viscosity with increasing shear rate. When shear rate was below 100 s^−1^, the apparent viscosity of the samples sharply decreased. This phenomenon indicates that the fluid exhibits significant shear thinning behavior in the low-shear-rate range. An increase in shear rate destroys the protein aggregation network structure and oil droplet structure in soybean flour, reduces flow resistance, and thereby results in a decrease in viscosity. The viscosity of SF was the lowest. After the ball milling combined with GABA treatment, the viscosity of BGSF further increased. And the viscosity also increased with an increase in GABA concentration. This may be because ball milling changed the particle morphology and size of the soybean flour, increasing intermolecular interactions. The addition of GABA may have further modified the protein structure and molecular forces, leading to the observed increase in apparent viscosity [51]. In the food industry, shear thinning behavior is a critical parameter for technological processes such as mixing and pumping, as it reduces flow resistance and energy consumption under high-shear conditions [52]. This same rheological property is equally vital for consumer acceptance, as it enables the product to exhibit a thin and smooth mouthfeel.

The effect of the ball milling combined with GABA treatment on the shear rheological properties of BGSF is shown in Figure 6B,C. It can be seen that both G′ and G″ of the BGSF increased with an increase in frequency, and G″ remained higher than G′ throughout the entire frequency range. After the ball milling combined with GABA treatment, the modulus of BGSF significantly increased, indicating that the ball milling combined with GABA treatment can further improve the rheological properties of soybean flour solution.

### 3.17. The Antioxidant Capacity of BGSF Under Different Concentrations of GABA

**Figure 7:**
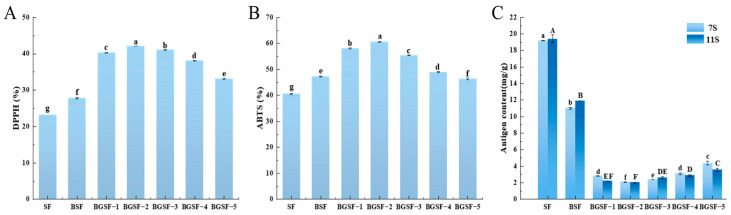
DPPH (**A**), ABTS (**B**), and antigen content (**C**) of soybean flour (SF), soybean flour treated by ball milling (BSF), and soybean flour under the ball milling combined with GABA treatment (BGSF). All results are presented as mean ± standard deviation (*n* = 3). Different letters in each column represent significant differences (*p* < 0.05). Capital letters indicate significant differences in 11S content, while lowercase letters indicate significant differences in 7S content (*p* < 0.05).

As shown in Figure 7A,B, the antioxidant capacity of BGSF at different concentrations of GABA was investigated. After the ball milling combined with GABA treatment, the DPPH and ABTS free radical scavenging rates of BGSF showed a trend of an initial rise followed by a decline. This may be due to GABA being a small molecule that has antioxidant activity. There are studies reporting that the accumulation of GABA contributes to the balance of gut microbiota, enhances the resistance of biomacromolecules and cellular organelles to reactive oxide species (ROS), and thereby inhibits exercise-induced fatigue and reduces the level of ROS [53]. It can be seen that BGSF−2 had the highest antioxidant capacity, with free radical scavenging rates of 42.13% and 60.61% for DPPH and ABTS, respectively. This may be due to ball milling altering the spatial structure of proteins, exposing more polar groups on their surfaces. This promotes the binding between the protein and GABA, leading to a further enhancement of its antioxidant capacity. Therefore, the ball milling combined with GABA treatment provides an effective strategy for improving the antioxidant properties of soybean flour, which is of great significance for enhancing the nutritional value of soybean flour in the food industry.

### 3.18. The Antigen Content of BGSF Under Different Concentrations of GABA

Figure 7C shows the antigen content of BGSF at different concentrations of GABA. The antigen content of BGSF significantly decreased after the addition of GABA. Additionally, as the GABA concentration increased, the antigen content showed a trend of first increasing and then decreasing. This indicates that the addition of GABA can effectively reduce the antigen content of soybean flour. Xing et al. [15] also confirmed that adding 0.40% GABA could reduce the antigen contents of 7S and 11S in SPI. Furthermore, the antigen contents of BGSF−2 were the lowest, at 2.09 mg/g and 2.01 mg/g, respectively. Compared with SF, the antigen contents of BGSF−2 decreased by 89.11% and 89.61%, respectively. This may be due to the intense mechanical forces generated by ball milling inducing conformational changes in the 11S and 7S globulins, thereby unfolding the protein structure and exposing the hidden linear epitopes within the proteins [54]. As a small molecule containing both amino and carboxyl groups, GABA possesses the ability to interact with proteins through multiple potential mechanisms, including hydrophobicity, hydrogen bonding, and electrostatic interactions [15]. These molecular interactions may alter the spatial conformation of the protein, leading to the coverage or destruction of both linear and conformational epitopes on the protein. Consequently, the allergenicity of soybean flour is reduced. This indicates that the synergistic effect of ball milling treatment and GABA can more effectively destroy or reduce the antigen structure in soybean flour, thereby reducing its immune activity.

## 4. Conclusions

This study developed a novel synergistic desensitization technology through the ball milling combined with GABA treatment, effectively overcoming the limitations of conventional single-mode processing approaches. Ball milling significantly reduced the particle size of soybean flour. When the material ball ratio was 1:14, BSF−14 exhibited the most optimal bulk density, wettability, dispersibility, and solubility. In addition, BSF−14 had the highest antioxidant capacity and the lowest antigen content. The ball milling combined with GABA treatment further improved the physicochemical properties of soybean flour on the basis of a single ball milling treatment. Additionally, the ball milling combined with GABA treatment further reduced antigen content. The antigen contents of β-conglycinin and glycinin in BGSF−2 decreased by 89.11% and 89.61%, respectively, compared to the control group. Furthermore, BGSF−2 exhibited the highest solubility and free radical scavenging rate, indicating that the ball milling combined with GABA treatment provides an effective strategy for enhancing the functional properties of soybean flour.

In conclusion, this study confirms that GABA can serve as an effective desensitizing substance in food, providing valuable innovative strategies and methods for the production of low-antigenic soybean flour. However, in the food industry, this combined technology needs to overcome challenges such as the cost of ball milling and the impact of spray-drying parameters following GABA addition on the sensory quality of soybean flour products before it is widely adopted. In addition, future research will focus on animal experiments and clinical trials to further evaluate and validate these findings. A sensory evaluation will be incorporated into the assessment system, aiming to develop a low-antigen functional soybean flour food formulation.

## Figures and Tables

**Table 1:** The color of soybean flour (SF), soybean flour treated by ball milling (BSF), and soybean flour under the ball milling combined with GABA treatment (BGSF).

Sample	L*	a*	b*
SF	82.74 ± 0.04 ^e^	1.97 ± 0.03 ^b^	27.42 ± 0.17 ^b^
BSF	84.72 ± 0.05 ^d^	2.56 ± 0.10 ^a^	28.41 ± 0.07 ^a^
BGSF−1	86.35 ± 0.04 ^b^	1.92 ± 0.06 ^b^	26.79 ± 0.18 ^c^
BGSF−2	86.56 ± 0.03 ^a^	1.82 ± 0.01 ^c^	26.57 ± 0.01 ^d^
BGSF−3	86.57 ± 0.07 ^a^	1.80 ± 0.02 ^c^	26.33 ± 0.07 ^e^
BGSF−4	86.29 ± 0.06 ^b^	1.75 ± 0.04 ^cd^	26.23 ± 0.01 ^e^
BGSF−5	85.94 ± 0.04 ^c^	1.66 ± 0.03 ^d^	26.19 ± 0.04 ^e^

Note: Means with different superscript letters within the same column for each parameter are significantly different (*p* < 0.05).

## Data Availability

The original contributions presented in the study are included in the article; further inquiries can be directed to the corresponding authors.
